# Effectiveness of behavioural economics‐based financial incentives and social feedback on glycaemic control and physical activity in adults with newly diagnosed type 2 diabetes: A randomised control trial

**DOI:** 10.1111/dom.70541

**Published:** 2026-02-09

**Authors:** Cheryl W. Y. Lai, Harley H. Y. Kwok, Jiaxi Ye, Carmen S. Ng, Pang Fai Chan, David V. K. Chao, Tsun‐kit Chu, Matthew M. H. Luk, Ming‐chuen Sin, Jenny H. L. Wang, Man‐kin Wong, Gabriel M. Leung, Helen Zhi, Parco M. Siu, Jianchao Quan

**Affiliations:** ^1^ School of Public Health, LKS Faculty of Medicine The University of Hong Kong Hong Kong; ^2^ School of Public Health Imperial College London London UK; ^3^ Department of Family Medicine & Primary Health Care United Christian Hospital Hong Kong; ^4^ Department of Family Medicine & Primary Health Care Tuen Mun Hospital Hong Kong; ^5^ Department of Family Medicine & Primary Health Care Hong Kong West Cluster Hong Kong

**Keywords:** behavioural economics, clinical trial, diabetes, exercise, incentives, physical activity

## Abstract

**Aims:**

To assess the effectiveness of behavioural economics‐based financial incentive and social comparison feedback in adults with newly diagnosed type 2 diabetes.

**Materials and Methods:**

We conducted a pragmatic randomised control trial with 6‐month intervention and 3‐month post‐intervention follow‐up. Participants were randomised to three groups: financial incentive (FI), financial incentive and social comparison feedback (FS), and control. Intervention groups received loss‐framed financial incentives to meet personalised weekly step‐count targets, with the FS group additionally receiving peer encouragement. We performed intention‐to‐treat analysis with multiple imputation and generalised estimating equations (GEE).

**Results:**

Among 99 adults newly diagnosed with type 2 diabetes, mean HbA1c at baseline in the control, FI, and FS groups were as follows: 52.5 (NSGP: 7%) (SD: 5.4), 52.9 (7%) (4.1), and 54.5 (7.1%) (4.9) mmol/mol. There were no significant decreases in HbA1c, weight, cholesterol or triglycerides in the incentive groups versus control at 6 or 9 months. There were significant increases in step count and physical activity at 6 months compared to control (FI: +1283 steps/day, *p* < 0.001; FS: +1545 steps/day, *p* < 0.001; FS group +774 MET‐minutes/week, *p* < 0.001).

**Conclusions:**

Financial incentive and social comparison feedback improved physical activity in people newly diagnosed with type 2 diabetes but were insufficient to achieve a significant improvement in glycaemic control.

## 1 INTRODUCTION

Chronic non‐communicable diseases such as type 2 diabetes mellitus impose a heavy health and economic burden globally. Much like the rest of the world, the prevalence of type 2 diabetes among adults in Hong Kong and mainland China has already exceeded 10%.^1^ Worryingly, the prevalence is forecasted to increase based on current epidemiological trends with the total annual cost of diabetes predicted to reach US$460.4 billion by 2030 in China alone.^2^

Individual levels of physical activity are strongly associated with the prevention and management of type 2 diabetes.^3^ A 20% increase in daily step count (approximately 1190 extra steps/day) over 12 months can result in a significant decrease in HbA1c for people with type 2 diabetes and hypertension.^4^ Despite robust evidence showing a healthy lifestyle is beneficial for glycaemic control, people with diabetes typically report significantly lower levels of physical activity than people without diabetes.^5^ Over 56% of the Hong Kong population do not meet the World Health Organisation recommendations for physical activity.^6^ An intensive behavioural lifestyle intervention program is strongly recommended for people with newly diagnosed type 2 diabetes to control diabetes in clinical best practice guidelines in Hong Kong, UK, and the United States.^7^, ^8^, ^9^ According to the Standards of Care, adults with type 2 diabetes are recommended to engage in at least 150 min of moderate‐to‐vigorous‐intensity activity per week for better glycaemic and weight control.^10^

Step count is a practical measure for monitoring physical activity with 10 000 steps per day considered a reasonable target for adults.^11^ Therefore, setting 10 000 steps as a maximum target in this study, combined with personalised step goals, provides a realistic and evidence‐based approach for progressively increasing physical activity in people with type 2 diabetes.^12^

Insights from behavioural economics incorporate psychological elements in understanding the economic decision‐making processes of individuals^13^ and such techniques from behavioural economics could be leveraged in designing incentives.^14^, ^15^ For example, individuals are more motivated by framing rewards as losses rather than gains; this loss aversion can be incorporated in the incentives by offering payment upfront with money deducted if targets are unmet. Frequent individualised feedback with appropriate framing of the incentive can reinforce behavioural change. Non‐financial techniques can also be leveraged, such as feedback of performance relative to peers (rank/percentile), social influence, priming, and gamification.

Although several studies based on behavioural economics have been shown to improve diabetes prevention and self‐management,^16^ these studies have generally used incentives other than loss‐framed approaches for physical health or habit formation, and the effects of such incentives on the early stages of diseases remain unclear. Previous research has shown that loss‐framed financial incentives can effectively help with habit formation though they lack clinical or laboratory measures of disease management. For instance, a randomised controlled trial of 105 patients with ischaemic heart disease found that loss‐framed financial incentives combined with personalised goal setting significantly increased daily step counts over 16 weeks, compared to controls.^17^ Similarly, in a trial of 281 overweight and obese adults, only the loss‐incentive group achieved a significantly higher mean proportion of days meeting the 7000‐step goal during a 13‐week intervention, compared to control, gain‐incentive, and lottery‐incentive groups.^18^

Developing new habits may be best facilitated by presenting incentives at opportune moments when an individual is most likely to act, such upon receipt of a new diagnosis. Early intervention in diabetes and pre‐diabetes is crucial, as prompt lifestyle modification can improve long‐term outcomes and reduce the risk of complications. A multidisciplinary task force of experts recommends management should be initiated as soon as risk factors or early disease are recognised, ideally before downstream complications develop.^19^ Achieving therapeutic targets early through diet, physical activity, and medication when appropriate could help halt the progression of dysglycaemia.^19^ In individuals with pre‐diabetes, early treatment may even promote a return to normoglycaemia.^20^ This underscores the importance of timely behavioural modification as the foundation of diabetes prevention and management.

Despite the global need for early action, most existing trials of behavioural economic interventions have focused on Western settings, where cultural attitudes to risk perception and risk‐taking behaviour may differ. With an estimated 233 million individuals living with diabetes in China in 2023,^21^ there is a critical need to evaluate these approaches in Chinese populations. This study therefore investigates the effect of behavioural economic intervention on glycaemic control by encouraging physical activity among Chinese adults newly diagnosed with type 2 diabetes.

## 2 MATERIALS AND METHODS

### 2.1 Study design

We conducted a three‐arm randomised controlled trial in adults newly diagnosed with type 2 diabetes. Participants wore a wearable fitness tracker for 9 months (6‐month intervention and 3‐month post‐intervention) to record their daily physical activity. Participants were randomly assigned to three groups on a 1:1:1 ratio: FI (financial incentive + standard care), FS (financial incentive and social comparison feedback + standard care), and control (standard care). During the 6‐month intervention period, daily step targets increased by 1000 steps each week from the participants' baseline levels to a maximum target of 10 000 steps per day. Participants were followed for 3 months after the cessation of interventions to assess any continued effects and the sustainability of lifestyle change. (ClinicalTrials.gov identifier: NCT04917926; registered 28 April 2021; https://linicaltrials.gov/study/NCT04917926).

### 2.2 Participants

Eligible participants were adults age 30–70 years at enrolment, newly diagnosed with type 2 diabetes within the past year, had HbA1c levels of 48–75 mmol/mol, willing to take blood tests, had access to a smartphone to track physical activity and receive text messages, physically mobile for the duration of the trial, and were residing in the community in Hong Kong, capable of providing informed consent, and able to communicate in English or Chinese. Newly diagnosed was defined as having received a diagnosis of type 2 diabetes within the past 12 months. Participants were excluded if they were on insulin for diabetes control, pregnant or breastfeeding, had surgery planned in the next 6 months, or had biological impairment or health conditions affecting the ability to walk, such as blindness, physical immobility, and paralysis.

Recruitment was conducted from June 2021 to February 2023 at four public general outpatient clinics and one district health centre for family medicine and primary care. Participants were identified during regular follow‐up visits at the participating centres. Researchers approached eligible individuals and used a screening questionnaire to confirm the date of diagnosis (self‐reported by participants), while recent HbA1c results were verified by checking the participants' actual medical records or laboratory reports. After providing written informed consent, participants answered a questionnaire on demographics, medication use, physical activity, and health‐related quality of life. Blood tests, weight, and follow‐up questionnaires were conducted at enrolment, 6 months, and 9 months (i.e., 3 months post‐intervention).

### 2.3 Sample size calculation

We aimed to enrol 261 participants (87 per group), based on an expected mean HbA1c difference of −0.6% (standard deviation 1.5%) from previous meta‐analyses.^22^, ^23^ Using an equal‐variance *t*‐test (significance level 0.05, power 80%), a sample size of 261 (87 per group) was required to allow for a 10% dropout rate. Calculations were performed using R (version 3.4). However, the study became underpowered due to two external factors: (1) recruitment was suspended due to Hong Kong's strict public health measures during the COVID pandemic, and (2) updated clinical guidelines recommending combined pharmacotherapy and lifestyle intervention immediately upon diagnosis of diabetes at lower blood glucose thresholds, rather than an initial 6 months of lifestyle modification, which significantly reduced the pool of eligible participants. The sample size of 99 participants has adequate power to detect a large effect size of 0.92, but only low‐moderate power to detect medium effects of 0.61 using guidelines of Cohen's *d* = 0.20, 0.50, and 0.80 to interpret observed effect sizes as small, medium, or large, respectively. These guidelines are recommended where field‐specific estimates are unknown, for example, where it might be unclear what would constitute a meaningful change in step count, or physical activity.

### 2.4 Interventions

All participants received their usual standard care including patient screening and education on diet, physical activity, and smoking cessation from a multidisciplinary team.^24^ Both the FI and FS intervention groups received a loss‐framed financial incentive and weekly feedback on performance for 6 months; with the FS group receiving additional social feedback. The financial incentive used a loss‐framed design based on the motivation of loss aversion and the endowment effect. Loss‐framed incentives have been found to be more effective at increasing physical activity than lottery‐framed incentives or gain‐framed incentives.^18^ Participants randomised to the intervention groups were credited with HK$1000 (US$128) at the beginning of the intervention period for the endowment effect. Following the measurement of baseline step counts in the first 2 weeks, HK$40 (US$5) was deducted each week (up to a total of 25 weeks) if they failed to achieve their personal weekly step target. Importantly, only those who consistently met their step targets throughout the intervention period retained the full amount. Participants who did not meet their targets forfeited part or all of the endowment and received no monetary reward at the end of the intervention. This amount of financial incentive is small compared to the expected direct medical cost of diabetes to the public sector in Hong Kong of HK$11 919 (US$1521) per year excluding any complications.^25^ Each week the participants were informed if they achieved their step target, the amount deducted, and their remaining financial balance. Feedback messages were positively framed and sent weekly via text to garner encouragement.

The social feedback were peer comparison and social support. During the intervention period, participants in the FS intervention group received weekly monitoring and individualised feedback via text message, which included the percentage of participants who achieved their target step counts that week as direct comparisons were not possible due to personalised target step counts. In addition to peer comparison, participant weekly performances were also shared with their nominated supporter to act as social support. These supporters could be their family members, friends, and colleagues.

All interventions were discontinued during the 3‐month post‐intervention follow‐up period. Participants in the control arm were not given any specific goals to achieve and did not receive any weekly feedback or messages.

### 2.5 Outcomes

The primary outcome was changes in glycated haemoglobin (HbA1c) levels at 6‐ and 9 months. Secondary outcomes included changes in daily step count, physical activity, weight, and lipid profile (cholesterol and triglycerides). Although all participants wore activity trackers with accelerometers (manufactured by LinkCo incorporating the G‐sensor SC7A20), this device does not measure the activity type or intensity. Therefore, physical activity was also assessed by metabolic equivalent [MET]‐minutes per week using the International Physical Activity Questionnaires [IPAQ].^26^

### 2.6 Randomisation

The random allocation process sequence was computer generated by an independent research assistant using R software program and concealed from the frontline team using tamper‐proof serial numbered opaque sealed envelopes. To avoid baseline unbalance, the randomisation was stratified by age (30–49, 50–70) and sex in block sizes of six. Laboratory and analysis staff were blinded to the allocation groups. An independent research assistant, uninvolved in the trial conduct, conducted interim analyses and safety monitoring.

### 2.7 Statistical analysis

Our analysis used an intention‐to‐treat (ITT) approach. For any missing data values, the researchers first rechecked the original records. In the analyses, we applied multiple imputation^27^ with 15 imputed datasets using the *mice* package in R and combined results according to Rubin's rules.^28^ The imputation predictors included HbA1c, LDL, triglycerides, weight, step count, and MET‐minutes at baseline, 6‐months, and 9‐months, age, and sex. Multiple imputation is commonly used in clinical trial data analysis to maximise both the available data and statistical power and is preferable to complete case analysis or single imputation methods.^29^

We fitted generalised estimating equation (GEE) models (baseline vs. 6 months and baseline vs. 9 months) to handle the repeated measurements with the following covariates: time, group (control, FI, and FS groups), group‐by‐time interaction, and baseline measurement of the outcome variables (daily step count, MET‐minute per week, HbA1c, weight, low‐density lipoprotein cholesterol, and triglycerides), age, and sex. Both autoregressive and exchangeable working correlation structures were tested in the GEE models. As the results were consistent, only the results from the autoregressive structure are reported here. We also conducted complete case analysis (*n* = 80 at 6 months; *n* = 79 at 9 months) using the GEE model. Statistical significance was set at *p*‐value <0.05. Analyses were performed using R (version 3.4).

## 3 RESULTS

### 3.1 Baseline characteristics of participants

**FIGURE 1:**
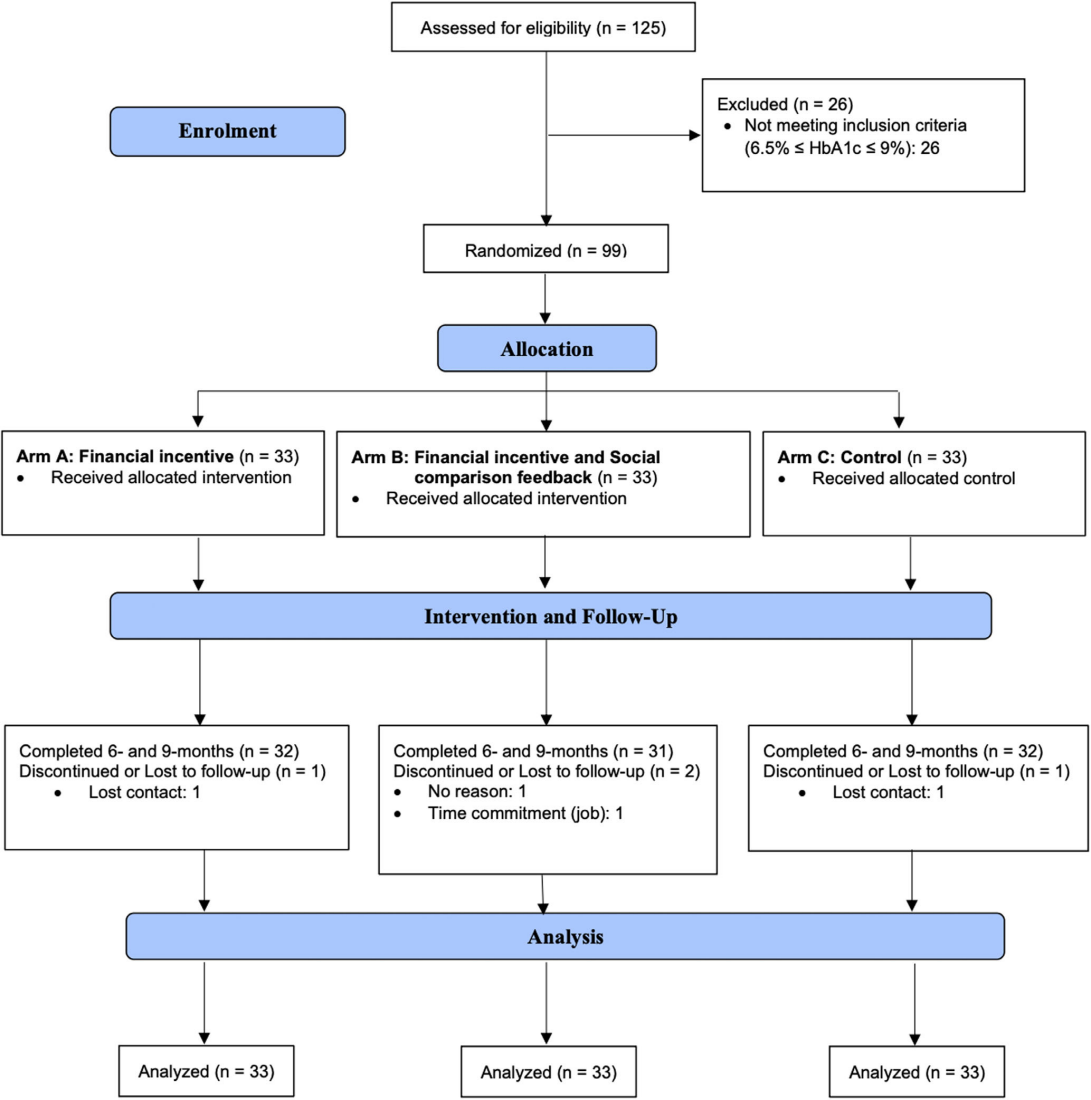
Flow diagram of study participants.

A total of 99 eligible persons provided informed consent and met the inclusion criteria (Figure 1); and were randomly assigned to FI (*n* = 33), FS (*n* = 33), and control (*n* = 33) groups. No significant differences in participant characteristics were found between groups at baseline (Table 1). At baseline, 64% of participants were overweight (BMI = 23–24.9) or obese (BMI ≥ 25) according to Hong Kong Department of Health thresholds (FI group: 67%; FS group: 58%, control: 73%), 45% of participants had HbA1c ≥53 mmol/mol (≥7%), exceeding the recommended HbA1c control target for non‐pregnant adults with type 2 diabetes (FI group: 25%; FS group: 64%; control: 27%).^30^

**TABLE 1:** Participant characteristics at baseline.

Characteristic	Control	Financial incentive	Financial incentive and social comparison feedback	*p*
*n*	33	33	33	
Male, *n* (%)	13 (39.4)	18 (54.5)	16 (48.5)	0.467^a^
Mean age (SD), years	56.9 (7.0)	54.1 (8.6)	54.5 (8.5)	0.311^b^
Mean height (SD), cm	160.3 (9.13)	164.5 (9.8)	162.3 (8.4)	0.173^b^
Mean weight (SD), kg	70.2 (18.5)	76.4 (20.0)	70.0 (16.6)	0.281^b^
Mean BMI (SD), kg/m^2^	27.0 (4.9)	28.0 (5.7)	26.3 (4.3)	0.398^b^
Mean HbA1c (SD), mmol/mol	52.48 (5.47)	52.87 (4.13)	54.46 (4.97)	0.222^b^
Mean low‐density lipoprotein (SD), mmol/L	2.68 (0.62)	2.71 (0.58)	3.03 (1.0)	0.129^b^
Mean triglyceride (SD), mmol/L	1.44 (0.76)	1.70 (1.46)	1.83 (2.74)	0.679^b^
Mean systolic blood pressure (SD), mmHg	132.2 (13.9)	134.4 (12.3)	131.6 (13.2)	0.667^b^
Mean diastolic blood pressure (SD), mmHg	82.1 (10.5)	83.9 (10.1)	81.5 (9.2)	0.589^b^
Median daily step count (IQR)	10 017 (4807)	9136 (4007)	9998 (6139)	0.268^b^
Mean physical activity level (SD), MET‐minutes/week	2208 (1633)	2181 (1430)	2318 (1758)	0.935^b^
On diabetes medication, *n* (%)	21 (63.6)	22 (66.7)	19 (57.6)	0.742^a^
Education level				
Primary	3 (9.1)	2 (6.1)	3 (9.1)	0.952^a^
Secondary	21 (63.6)	23 (69.7)	24 (72.7)	
Tertiary	8 (24.2)	7 (21.2)	6 (18.2)	
Refused	1 (3.0)	1 (3.0)	0	
Employment status				
Employed	18 (54.2)	17 (51.5)	18 (54.5)	0.906^a^
Homemakers	5 (15.2)	5 (15.2)	1 (3.0)	
Others	10 (30.3)	11 (33.3)	14 (42.4)	
Housing type				
Public rental	8 (24.2)	10 (30.3)	7 (21.2)	0.302^a^
Private permanent	21 (63.6)	18 (54.5)	24 (72.7)	
Subsidised home ownership	3 (9.1)	2 (6.1)	2 (6.1)	
Refused	1 (3.0)	3 (9.1)	0	
Monthly income (HK$)				
Below 20 000	18 (54.5)	16 (48.5)	15 (45.5)	0.940^a^
20 000 to 40 000	8 (24.2)	7 (21.2)	9 (27.3)	
Above 40 000	1 (3.0)	5 (15.2)	4 (12.1)	
Refused	6 (18.2)	5 (15.2)	5 (15.2)	

Abbreviations: IQR, interquartile range; SD, standard deviation.

^a^ Kruskal–Wallis test.

^b^ One‐way ANOVA.

### 3.2 Primary outcome

There were no significant changes in HbA1c for both intervention groups compared to the control at 6 and 9 months (Table 2). Compared to control, the mean difference in HbA1c at 6 months for FI was −0.95 mmol/mol (95% CI: −3.61 to 1.71) and for FS was −0.43 mmol/mol (95% CI: −3.27 to 2.41); and at 9 months for FI was 0.05 mmol/mol (−1.71 to 1.82) and for FS was −0.21 mmol/mol (−2.10 to 1.68). No significant differences were found in the complete case analysis.

**TABLE 2:** Primary and secondary outcomes at 6 months and 9 months (GEE model).

Outcome	Control (*n* = 33)	FI group (*n* = 33)	FS group (*n* = 33)	FI group versus control		FS group versus control		FS group versus FI group	
				Adjusted mean difference (95% Cl)	*p*	Adjusted mean difference (95% Cl)	*p*	Adjusted mean difference (95% Cl)	*p*
HbA1c, mean (SD), mmol/mol									
Baseline	52.48 (5.39)	52.87 (4.07)	54.46 (4.90)						
6 months	51.11 (9.23)	50.52 (4.91)	52.71 (6.32)	−0.95 (−3.61 to 1.71)	0.483	−0.43 (−3.27 to 2.41)	0.767	0.62 (−2.01 to 3.25)	0.645
9 months	50.80 (6.31)	51.25 (5.20)	52.34 (7.13)	0.05 (−1.71 to 1.82)	0.952	−0.21 (−2.10 to 1.68)	0.828	−0.23 (−2.03 to 1.57)	0.800
Daily step count, mean (SD)									
Baseline	10 805 (4250)	9599 (3157)	11 330 (5368)						
6 months	11 075 (4289)	11 165 (3461)	13 167 (4802)	1283 (694 to 1871)	**<0.001**	1545 (861 to 2229)	**<0.001**	238 (−313 to 788)	0.398
9 months	10 553 (3803)	9835 (3655)	11 672 (5481)	236 (−113 to 585)	0.184	308 (−11 to 626)	0.058	30 (−382 to 442)	0.885
MET, mean (SD), MET‐minutes/week									
Baseline	2208 (1609)	2181 (1410)	2318 (1733)						
6 months	2516 (1469)	2590 (1586)	3394 (1856)	108 (−59 to 275)	0.203	774 (539 to 1008)	**<0.001**	664 (434 to 893)	**<0.001**
9 months	3059 (1399)	3208 (1691)	3143 (1416)	107 (39 to 174)	**0.002**	−16 (−74 to 42)	0.587	−106 (−176 to −37)	**0.003**
Weight, mean (SD), kg									
Baseline	70.16 (18.20)	76.39 (19.70)	70.04 (16.32)						
6 months	70.29 (20.86)	74.67 (18.60)	69.46 (17.26)	−1.37 (−5.00 to 2.27)	0.461	−0.92 (−4.73 to 2.89)	0.637	0.57 (−2.79 to 3.94)	0.739
9 months	67.93 (18.68)	73.19 (19.10)	69.36 (17.05)	0.07 (−1.75 to 1.89)	0.939	0.82 (−1.52 to 3.16)	0.494	0.79 (−1.31 to 2.90)	0.460

Abbreviations: FI, financial incentive group; FS, financial incentive and social comparison feedback group; GEE, generalised estimating equation; LDL, low‐density lipoprotein; MET, metabolic equivalent; SD, standard deviation.

### 3.3 Secondary outcomes

**FIGURE 2:**
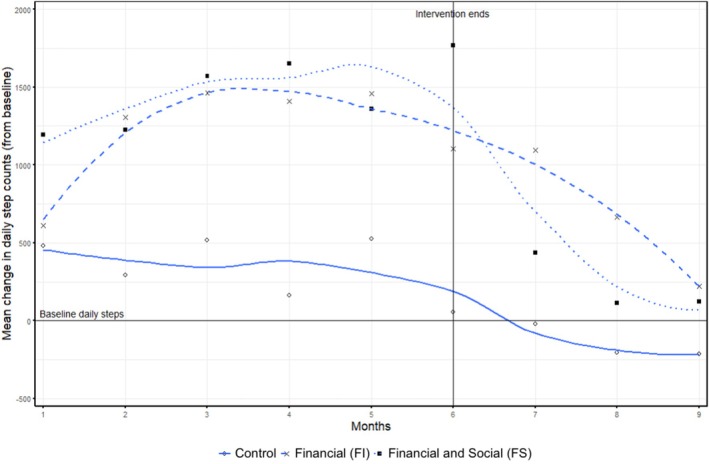
Changes in daily step count over study period by group.

Both intervention groups showed a significant increase in daily step count at the end of the 6‐month intervention period compared to control (mean differences: FI group: +1283 steps, *p* < 0.001; FS group: +1545 steps, *p* < 0.001) (Table 2). There was a significant increase in physical activity (MET‐minutes per week) compared to control in the FS group (mean difference: +774 MET‐min/week, *p* < 0.001), but not in the FI group (+108 MET‐min/week, *p* = 0.203). Notably, during the 3‐month post‐intervention period, all groups showed drops in step count, particularly the control and FS groups (Figure 2). At 9 months, only the FI group showed significant increases in MET‐minutes per week compared to control (mean difference: +107 MET‐min/week, *p* = 0.002) while a slight decrease was found in the FS group compared to control (−16 MET‐min/week, *p* = 0.587).

At 6 months, there were some improvements in weight and lipid profile in both intervention groups, but no significant differences compared to control (Table 2 and Appendix Table 2). Non‐significant improvements in weight loss were found for both intervention groups compared to control; mean differences of −1.37 kg (95% CI: −5.00 to 2.27) in the FI group and −0.92 kg (95% CI: −4.73 to 2.89) in the FS group. Non‐significant improvements in lipid profile were found for both intervention groups. Compared to control at 6 months, the mean difference in low‐density lipoprotein (LDL) cholesterol was −0.10 mmol/L (95% CI: −0.83 to 0.62) in the FI group and −0.22 mmol/L (95% CI: −1.08 to 0.63) in the FS group, and the mean difference in triglycerides was −0.10 mmol/L (95% CI: −1.04 to 0.85) in the FI group and −0.55 mmol/L (95% CI: −1.90 to 0.81) in the FS group. At 9 months, there remained small non‐significant improvement in weight and lipid profile in the intervention groups compared to control.

The complete case analysis showed no significant changes in step count and MET at 6 months and 9 months (Appendix Table 3).

## 4 DISCUSSION

We conducted a three‐arm randomised controlled trial in adults newly diagnosed with type 2 diabetes to examine the effect of a behavioural economics‐based financial and social interventions on glycaemic control, physical activity, weight and lipid profile. We found improvements in glycaemic control (decreases in HbA1c levels) in all groups over the study period with no significant differences between intervention and control groups. Our study showed that financial interventions, with or without social support, encouraged adults with type 2 diabetes to improve their daily step count. Daily step count in both intervention groups dropped dramatically during the 3‐month follow‐up period. In the control group, the mean daily step count had a downward trend and dropped below its baseline after 6 months.

Our results show the potential feasibility of financial incentive and social comparison feedback to promote daily physical exercise for people with diabetes. Similar findings have been reported in other behavioural studies. For example, a behavioural study that investigated the effect of financial and social incentives on walking in older adults found that daily step count increased during the 16‐week intervention period.^31^ Another study in older Chinese adults showed that peer support via a mobile app‐based walking program improved individual step count by 408 steps/day after 3 months.^32^ Collectively, these findings suggest that walking interventions can effectively boost physical activity in the short term.

There were no significant improvements in HbA1c or other metabolic indicators, including weight, LDL‐cholesterol, and triglycerides, in both intervention groups during the study period. These results do not align with findings from other studies, such as the LOOK AHEAD study,^33^ which reported that more than 5% of weight loss was achieved in half of the participants with type 2 diabetes who received intensive lifestyle intervention over 8 years. Several factors may explain why the increased physical activity observed in our study did not translate into significant improvements in HbA1c or other metabolic outcomes. Effective diabetes management typically requires a combination of lifestyle interventions, including dietary modifications and smoking cessation, as well as appropriate pharmacological management.^34^ Our intervention focused primarily on step count, without addressing other lifestyle factors, which may have limited the impact on glycaemic control. Some participants may also have compensated for their increased activity by relaxing other healthy behaviours, such as diet. Walking was selected as the primary activity due to its accessibility and low skill requirement, with the goal of encouraging early lifestyle changes.^35^, ^36^ However, walking is generally less intense than other aerobic activities. For example, walking at 5 km/h yields 3.3 METs, compared to 11.5 METs for running at 11 km/h, and 8.0 METs for moderate cycling or swimming.^35^ Thus, the limited intensity and duration of walking may thus have been insufficient to produce significant metabolic effects. A systematic review on the effect of physical activity showed that high‐intensity training led to a significantly greater reduction in HbA1c level compared to that of moderate‐intensity training.^37^ Although walking may be beneficial for glycaemic control, its effect might be less efficacious than more intense resistance exercises.^38^ Thus, incorporating muscle‐strengthening exercises could potentially provide additional benefits.^35^

Adherence and sustainability of physical activity also play critical roles. Although step counts increased significantly during the 6‐month intervention, a noticeable decline was seen 3 months after the intervention, suggesting that incentives were the primary motivator. The decline in physical activity in the FS group after social feedback ended further underscores the importance of ongoing social reinforcement. A meta‐analysis of walking interventions in type 2 diabetes showed that supervised walking significantly reduced HbA1c, while non‐supervised walking was only effective when combined with motivational strategies^39^ such as regular phone calls from peer counsellors^40^ or peer‐led self‐management programs.^41^ Together, these findings underscore that sustaining behaviour change after the intervention period remains a major challenge, especially when the motivating effects of incentives diminish. For example, in two randomised controlled trials, loss‐framed financial incentives significantly increased physical activity during the intervention periods, but these effects diminished after interventions were withdrawn, with step counts declining during follow‐up.^17^, ^18^ These findings indicate that although loss‐framed financial incentives can boost short‐term physical activity, additional strategies are needed to support long‐term behaviour change. For example, sustained supervision and continuous social reinforcement appear essential for maintaining long‐term improvements in health behaviours.

Habit formation is a complex process influenced by multiple factors. Although the intervention period in this study was relatively long, automaticity in health behaviours does not develop uniformly across individuals and behaviours. A systematic review found that the median time required to form a healthy habit ranged from 59 to 66 days, but also highlighted that habit type, context stability, and personal involvement in goal selection all affect the strength and speed of habit formation.^42^ In our study, the abrupt removal of financial incentive and social support may have led to a sudden loss of motivation for some participants and a lack of sustained behaviour change.

The similar outcomes observed between FS and FI may be attributed to the social incentive design, which relied on normative feedback rather than strong competitive or reputational elements. Prior research suggests that the impact of social incentives varies with context.^43^ For example, competition with a higher performer or cooperation with a lower performer can both enhance performance, but their effects depend on the nature of the interaction.^43^ Notably, competing with a more skilled opponent has been shown to increase performance, but also stress and heart rates.^43^ In our study, the use of passive social comparison alone may have limited the incremental benefit of the social component beyond financial incentives.

To better sustain long‐term behavioural change, future clinical and public health interventions could use a tapered or step‐down approach to incentives, rather than abrupt withdrawal, to help transition participants from external motivation to intrinsic habit. Ongoing low‐intensity support such as digital reminders, periodic social feedback, and peer support could also reinforce new behaviours after the main intervention. While optimal diabetes control often requires comprehensive, multidisciplinary care involving professionals such as family physicians, nurses, dietitians, optometrists, and podiatrists,^44^ these strategies can guide the design of long‐term incentive programs and inform public health policies to promote sustained improvements in physical activity and metabolic health among people with diabetes.

### 4.1 Strengths

Our study used a randomised control trial to examine the effect of increased physical activity incentivised through behavioural economic interventions on glycaemic control. Participants were asked to provide their daily step count every week using fitness trackers to allow accurate and consistent measurements of daily step counts. This study also used laboratory measures such as HbA1c, LDL‐cholesterol, and triglycerides as objective indicators of glycaemic and lipid control to monitor disease management over the study period.

### 4.2 Limitations

Our study has several limitations. Firstly, the sample size was insufficient to detect low and medium effects and was thus underpowered for improvements in HbA1c, weight, and lipid profile to reach statistical significance. As a result, smaller but clinically meaningful effects may not be detectable. Secondly, there were instances of missing data, including missed blood tests at follow‐up and incomplete step count data due to non‐wearing of the device. Although the use of a uniform wearable device enhanced consistency, identification of non‐wear periods often relied on participant self‐report. These limitations were addressed analytically by accounting for missing values in the data analysis. Thirdly, changes in clinical practice for diabetes management to prioritise early initiation of pharmacotherapy upon diagnosis of diabetes likely impacted the results. At baseline, over 57% of participants in each group had already started medications such as metformin, which may have limited the scope for further improvement. Fourthly, public health measures during the COVID pandemic hampered study recruitment and restricted opportunities for outdoor physical activity. Finally, the maximum daily step count goal for participants was set at 10 000, which is one of the Hong Kong government recommendations to promote health. Yet nearly half (45%) of the participants newly diagnosed with diabetes have already reached the 10 000 daily step count goal at the beginning of the study, which may have limited the potential for further increases in activity and the effectiveness of the intervention.

## 5 CONCLUSIONS

We found both financial incentive and social comparison feedback improved step counts and physical activity in people newly diagnosed with type 2 diabetes. However, these interventions were insufficient to significantly improve glycaemic control, weight, cholesterol, or triglycerides.

## AUTHOR CONTRIBUTIONS

Jianchao Quan conceived the study and designed the study methodology with Helen Zhi and Parco M. Siu. Cheryl W. Y. Lai, Harley H. Y. Kwok, and Jiaxi Ye collected the data and conducted the project. Pang Fai Chan, David V. K. Chao, Tsun‐kit Chu, Matthew M. H. Luk, Ming‐chuen Sin, Jenny H. L. Wang, and Man‐kin Wong assisted with the data collection. Cheryl W. Y. Lai and Carmen S. Ng conducted the analysis and data visualisation. All authors interpreted the data. Jianchao Quan supervised the study. Jianchao Quan, Gabriel M. Leung, Helen Zhi and Parco M. Siu acquired funding. Cheryl W. Y. Lai, Harley H. Y. Kwok, and Jianchao Quan drafted and revised the manuscript. All authors reviewed and approved the submitted version.

## FUNDING INFORMATION

Research Grants Council, University Grants Committee, Hong Kong Special Administrative Region, China (17607619). The study funder was not involved in the design of the study; the collection, analysis, and interpretation of data; writing the report; and did not impose any restrictions regarding the publication of the report.

## CONFLICT OF INTEREST STATEMENT

The authors declare no conflicts of interest.

## ETHICS STATEMENT

The study was approved by the Institutional Review Boards of the Hospital Authority (HKU‐Hong Kong West [UW 19‐605], New Territories West [NTWC/REC/20031], and Kowloon Central/Kowloon East [KC/KE‐21‐0315/ER‐1]). Written informed consent was obtained from all participants.

## CONSENT

Consent forms are available upon request.

## Supporting information


**Appendix S1.** Supplementary Appendix.

**Data S1.** Reporting checklist for randomised trial.

**Data S2.** Supporting Information.

## Data Availability

The data that support the findings of this study are available on request from the corresponding author. The data are not publicly available due to privacy or ethical restrictions.
