# Supplementary material for: Effectiveness of behavioural economics‐based financial incentives and social feedback on glycaemic control and physical activity in adults with newly diagnosed type 2 diabetes: A randomised control trial
Source: Diabetes Obes Metab. 2026 Feb 9;28(5):3644–54. doi: 10.1111/dom.70541 (PMC13071219; doi:10.1111/dom.70541)
Supplement: Supplementary file 1 — Appendix S1. Supplementary Appendix. [file DOM-28-3644-s001.docx]

# APPENDIX

## Appendix Table 1. Missing values by group

| **Variables** | **Financial incentive** | **Financial incentive and Social comparison feedback** | **Control** |
| --- | --- | --- | --- |
| **At 6 months** |  |  |  |
| HbA1c | 5 | 5 | 2 |
| Daily step counts | 1 | 2 | 4 |
| MET | 2 | 2 | 1 |
| Weight | 3 | 2 | 3 |
| LDL-cholesterol | 5 | 5 | 2 |
| Triglycerides | 5 | 5 | 2 |
| **At 9 months** |  |  |  |
| HbA1c | 5 | 5 | 3 |
| Daily step counts | 3 | 5 | 2 |
| MET | 1 | 2 | 2 |
| Weight | 1 | 5 | 3 |
| LDL-cholesterol | 5 | 5 | 3 |
| Triglycerides | 5 | 5 | 3 |

MET, metabolic equivalent.

## Appendix Table 2. Secondary Outcomes (Lipid profile) at 6-months and 9-months using GEE model

| **Outcome** | **Control  (n = 33)** | **FI Group (n = 33)** | **FS Group (n = 33)** | **FI Group vs Control** | | **FS Group vs Control** | | **FS Group vs FI Group** | |
| --- | --- | --- | --- | --- | --- | --- | --- | --- | --- |
|  |  |  |  | **Adjusted Mean Difference**  **(95% Cl)** | **P Value** | **Adjusted Mean Difference**  **(95% Cl)** | **P Value** | **Adjusted Mean Difference**  **(95% Cl)** | **P Value** |
| **LDL-Cholesterol, mean (SD), mmol/L** | | |  |  |  |  |  |  |  |
| Baseline | 2.68 (0.61) | 2.71 (0.57) | 3.03 (0.99) |  |  |  |  |  |  |
| 6 months | 2.80 (0.73) | 2.72 (0.52) | 2.92 (0.83) | −0.10 (−0.83 to 0.62) | 0.786 | −0.22 (−1.08 to 0.63) | 0.609 | −0.12 (−0.98 to 0.75) | 0.793 |
| 9 months | 2.75 (0.86) | 2.86 (0.55) | 2.87 (0.84) | 0.05 (−0.48 to 0.59) | 0.847 | −0.11 (−0.72 to 0.50) | 0.721 | −0.16 (−0.78 to 0.47) | 0.621 |
| **Triglycerides, mean (SD), mmol/L** | | |  |  |  |  |  |  |  |
| Baseline | 1.44 (0.75) | 1.70 (1.44) | 1.83 (2.70) |  |  |  |  |  |  |
| 6 months | 1.55 (0.74) | 1.71 (1.62) | 1.40 (0.72) | −0.10 (−1.04 to 0.85) | 0.841 | −0.55 (−1.90 to 0.81) | 0.428 | −0.46 (−1.88 to 0.96) | 0.527 |
| 9 months | 1.59 (1.04) | 1.65 (1.19) | 1.48 (0.87) | −0.10 (−0.83 to 0.63) | 0.795 | −0.25 (−1.14 to 0.64) | 0.588 | −0.16 (−1.10 to 0.79) | 0.743 |

FI, financial incentive group; FSI, financial incentive and social comparison feedback group; GEE, Generalized Estimating Equation.

## Appendix Table 3. Primary and Secondary Outcomes at 6-months and 9-months using complete case analysis (GEE model)

| **Outcomes** | **Control  (n = 33)** | **FI Group (n = 33)** | **FS Group (n = 33)** | **FI Group vs Control** | | **FS Group vs Control** | |
| --- | --- | --- | --- | --- | --- | --- | --- |
|  |  |  |  | **Adjusted Mean Difference (95% Cl)** | **P Value** | **Adjusted Mean Difference (95% Cl)** | **P Value** |
| **Mean HbA1c (SD), mmol/mol** | |  |  |  |  |  |  |
| Baseline | 51.83 (5.32) | 52.56 (3.65) | 54.38 (4.97) |  |  |  |  |
| 6 months | 48.93 (5.21) | 50.01 (4.29) | 53.13 (6.43) | 0.35 (−2.60 to 3.30) | 0.816 | 1.64 (−1.86 to 5.14) | 0.358 |
| 9 months | 51.12 (6.49) | 50.74 (4.95) | 52.32 (7.91) | −0.06 (−1.65 to 1.54) | 0.946 | −0.17 (−2.20 to 1.85) | 0.866 |
| **Mean daily step count (SD), steps** | | | | | | | |
| Baseline | 10763 (3982) | 9799 (3422) | 11575 (5424) |  |  |  |  |
| 6 months | 11363 (4152) | 11223 (3464) | 13701 (4175) | 819 (−845 to 2483) | 0.335 | 1523 (−393 to 3438) | 0.119 |
| 9 months | 10079 (3627) | 10264 (3034) | 12549 (5927) | 376 (−312 to 1064) | 0.284 | 345 (−869 to 1559) | 0.577 |
| **Mean MET (SD), MET-minutes/week** | | |  |  |  |  |  |
| Baseline | 2375 (1659) | 2201 (1329) | 2384 (1777) |  |  |  |  |
| 6 months | 2681 (1405) | 2700 (1668) | 3536 (1867) | 190 (−838 to 1218) | 0.717 | 848 (−162 to 1858) | 0.100 |
| 9 months | 2918 (1420) | 3366 (1644) | 3151 (1356) | 279 (−128 to 687) | 0.179 | 36 (−418 to 490) | 0.875 |
| **Mean weight (SD), kg** | |  |  |  |  |  |  |
| Baseline | 65.86 (11.16) | 76.63 (17.56) | 69.56 (16.25) |  |  |  |  |
| 6 months | 64.52 (10.91) | 74.70 (18.23) | 68.91 (16.82) | −0.69 (−2.09 to 0.72) | 0.340 | 0.67 (−0.61 to 1.96) | 0.304 |
| 9 months | 68.67 (19.77) | 70.86 (17.17) | 66.77 (13.16) | −0.53 (−1.31 to 0.25) | 0.186 | 0.32 (−0.35 to 1.00) | 0.348 |
| **Mean low-density lipoprotein (SD), mmol/L** | | |  |  |  |  |  |
| **Baseline** | **2.72 (0.64)** | 2.69 (0.59) | 3.11 (1.04) |  |  |  |  |
| 6 months | 2.80 (0.76) | 2.68 (0.45) | 2.99 (0.88) | −0.10 (−0.35 to 0.15) | 0.439 | −0.21 (−0.63 to 0.20) | 0.306 |
| 9 months | 2.69 (0.84) | 2.85 (0.53) | 2.82 (0.92) | 0.047 (−0.10 to 0.20) | 0.531 | −0.16 (−0.38 to 0.07) | 0.167 |
| **Mean triglyceride (SD), mmol/L** | |  |  |  |  |  |  |
| Baseline | 1.41 (0.80) | 1.83 (1.57) | 1.97 (3.01) |  |  |  |  |
| 6 months | 1.48 (0.74) | 1.79 (1.78) | 1.39 (0.61) | −0.12 (−0.61 to 0.37) | 0.626 | −0.66 (−1.75 to 0.43) | 0.236 |
| 9 months | 1.64 (1.09) | 1.67 (1.30) | 1.56 (0.94) | −0.15 (−0.45 to 0.16) | 0.340 | −0.34 (−0.87 to 0.20) | 0.218 |

FI, financial incentive group; FSI, financial incentive and social comparison feedback group; GEE, Generalized Estimating Equation; LDL-cholesterol, low-density-lipoprotein cholesterol; MET, metabolic equivalent; SD, standard deviation.
