# Supplementary material for: Effectiveness of behavioural economics‐based financial incentives and social feedback on glycaemic control and physical activity in adults with newly diagnosed type 2 diabetes: A randomised control trial
Source: Diabetes Obes Metab. 2026 Feb 9;28(5):3644–54. doi: 10.1111/dom.70541 (PMC13071219; doi:10.1111/dom.70541)
Supplement: Supplementary file 3 — Data S2. Supporting Information. [file DOM-28-3644-s002.docx]

Effectiveness of behavioural economics-based incentives on glycaemic control and lifestyle in adults with newly diagnosed type 2 diabetes: a randomized control trial

**STUDY PROTOCOL**

Version 5

Date: Sep 6, 2022

**School of Public Health, The University of Hong Kong**

Dr Jianchao Quan (PI)

Prof. Gabriel Leung, Dr Parco Siu, Dr Helen Zhi

**Site Co-Investigators**

New Territories West Cluster: Dr Tsun-kit Chu, Dr Man-kin Wong

Hong Kong West Cluster: Dr Ming-chuen Sin, Dr Jenny Hua Li Wang

Kowloon East Cluster: Dr David Vai Kiong Chao, Dr Pang Fai Chan, Dr Man Hei Matthew Luk

**Project centre:**

School of Public Health, Li Ka Shing Faculty of Medicine, The University of Hong Kong
UB/F, Patrick Manson Building, 7 Sassoon Road, Hong Kong

Email: [jquan@hku.hk](mailto:jquan@hku.hk)

Tel: 3917 9909

Table of Contents

[SUMMARY OF PROTOCOL CHANGES 4](#_Toc219214380)

[1 General Information 5](#_Toc219214381)

[2 Background Information 5](#_Toc219214382)

[3 Aims and Objectives 6](#_Toc219214383)

[3.1 Primary objective 6](#_Toc219214384)

[3.2 Secondary objectives 7](#_Toc219214385)

[4 Study Design 7](#_Toc219214386)

[4.1 Participant enrolment 8](#_Toc219214387)

[4.2 Informed consent 8](#_Toc219214388)

[4.3 Enrolment interview 9](#_Toc219214389)

[4.4 Collection of blood samples 9](#_Toc219214390)

[4.5 Randomization 9](#_Toc219214391)

[4.6 Follow-up assessments 11](#_Toc219214392)

[4.7 Incentives 11](#_Toc219214393)

[5 Selection and Withdrawal of Subjects 11](#_Toc219214394)

[5.1 Subject inclusion criteria 11](#_Toc219214395)

[5.2 Subject exclusion criteria 12](#_Toc219214396)

[5.3 Subject withdrawal criteria 12](#_Toc219214397)

[6 Interventions to be Administered 12](#_Toc219214398)

[6.1 Medications permitted and not permitted before and/or during the study 13](#_Toc219214399)

[6.2 Stopping rule 14](#_Toc219214400)

[7 Outcome Measures 14](#_Toc219214401)

[7.1 Primary outcome 14](#_Toc219214402)

[7.2 Secondary outcomes 14](#_Toc219214403)

[7.3 Methods for assessing and analysing of efficacy parameters 14](#_Toc219214404)

[8 Assessment of Safety 15](#_Toc219214405)

[9 Statistical analysis 15](#_Toc219214406)

[9.1 Statistical methods 15](#_Toc219214407)

[9.2 Procedure for accounting for any missing data 15](#_Toc219214408)

[9.3 Procedure for reporting any deviations from the original statistical plan 16](#_Toc219214409)

[9.4 Selection of participants to be included in the analyses 16](#_Toc219214410)

[9.5 Sample size justification 16](#_Toc219214411)

[10 Direct Access to Source Data 16](#_Toc219214412)

[11 Quality Control and Quality Assurance 16](#_Toc219214413)

[12 Ethics 17](#_Toc219214414)

[13 Data Handling and Record Keeping 17](#_Toc219214415)

[14 Financing 18](#_Toc219214416)

[15 Publication Policy 18](#_Toc219214417)

[16 Supplements 18](#_Toc219214418)

[17 References 18](#_Toc219214419)

# SUMMARY OF PROTOCOL CHANGES

Changes to the protocol since version 1.0 are summarized in the following table

| Number | Date | Type | Brief Details |
| --- | --- | --- | --- |
| 1 | 09/01/2020 | General Information | Inclusion of clinical site Co-Is |
| 2 | 09/01/2020 | Study Design | Simplify to two arm trial of intervention vs control |
| 3 | 20/01/2021 | Study Design | Pragmatic study design: eligibility criteria, sample size |
| 4 | 06/05/2021 | General Information;  Study Design | Inclusion of new clinical site Co-I;  Original three arm trial plus pragmatic study design; modified eligibility criteria |
| 5 | 06/09/2022 | Study Design | Adding recruitment sites |

# General Information

**Principal Investigator**

Dr Jianchao QUAN
Clinical Assistant Professor, School of Public Health, The University of Hong Kong
Email: [jquan@hku.hk](mailto:jquan@hku.hk)
Tel: 3917 9909

**Co-investigators**

Prof. Gabriel Leung
Chair Professor, School of Public Health, The University of Hong Kong

Dr Parco Siu
Associate Professor, School of Public Health, The University of Hong Kong

Dr Helen Zhi
Scientific Officer, School of Public Health, The University of Hong Kong

***Site co-investigators***

Dr Jenny Hua Li Wang

Department of Family Medicine & Primary Health Care, Hong Kong West Cluster, Hospital Authority

Dr Tsun-kit Chu

Department of Family Medicine & Primary Health Care, Tuen Mun Hospital

Dr Man-Kin Wong

Madam Yung Fung Shee General Outpatient Clinic, New Territories West Cluster, Hospital Authority

Dr Ming-chuen Sin

Ap Lei Chau General Outpatient Clinic, Hong Kong West Cluster, Hospital Authority

# Background Information

Chronic non-communicable diseases such as diabetes mellitus are a major health expense for Hong Kong, and the rest of China. In Hong Kong and China, the prevalence of type 2 diabetes among the adults has exceeded 10% ^1^. Worryingly, this epidemic is projected to grow as over 35% of adults in China (388 million) have pre-diabetes ^2^. Modifiable lifestyle choices contribute to the growing epidemic of type 2 diabetes. Over 56% of adults in Hong Kong do not meet the World Health Organization recommendations for physical activity and over 79% consume less than the recommended five daily servings of fruit and vegetables ^3^. The first management step for a person with newly diagnosed type 2 diabetes is to adopt an intensive behavioural lifestyle intervention programme of healthy diet, physical activity and smoking cessation. This recommendation is consistent among clinical best practice guidelines in Hong Kong, UK and USA ^4–6^.

Insights from behavioural economics are promising ways for encouraging behaviour change. Increasingly employers and insurers are introducing behavioural incentives in health promotion programs to increase healthy choices such as physical activity ^7^. A number of techniques from behavioural economics could be leveraged in designing incentives ^8,9^. For example, individuals are more motivated by framing rewards as losses rather than gains; this loss aversion can be incorporated in the incentives by offering payment upfront with money taken away if targets are not met. Frequent individualised feedback with appropriate framing of the incentive could be used to reinforce the new behaviour. Non-financial techniques can also be leveraged such as feedback of performance relative to peers (rank/percentile), social influence, priming and gamification.

A recent review found a small number of trials of behavioural economic-based incentives on healthy behaviours such as weight loss, physical activity, and medication adherence ^10^. There is a lack of trials that measured clinical indicators such as glycaemic control (HbA1c), that are strong predictive of diabetes complications ^5^. Incentives based on behavioural economics have improved adherence to chronic disease management but have not been used at the early stages of diseases, where there lies an opportunity to delay or reverse the onset of diabetes. Developing new habits may be best facilitated by presenting incentives at moments when an individual is most likely to act, such as being informed of a new diagnosis of chronic disease. No trials have been conducted among the Chinese population with different cultural and social norms to risk compared to Western-settings, despite Chinese respondents being more risk-seeking than Americans ^11^.

# Aims and Objectives

The aim of this study is to test the effectiveness of incentives that use insights from behavioural economics to promote diabetes control and healthy behaviours among people newly diagnosed with type 2 diabetes.

## Primary objective

- To investigate the effect of financial and social incentives based on behavioural economic principles on glycaemic control at 6-months for people with newly diagnosed type 2 diabetes.

## Secondary objectives

- To examine the sustainability of glycaemic control at 9-months following the withdrawal of incentives for people with newly diagnosed type 2 diabetes.
- To study the effect of incentives based on behavioural economic principles on other health indicators including lipid profile, body weight/BMI, blood pressure, diet, physical activity, smoking, and delay of diabetes for people with newly diagnosed type 2 diabetes.

# Study Design

This study is a randomized controlled trial in adults aged 30-70 (at enrolment) with newly diagnosed type 2 diabetes over the study period of Jan 2020 to Dec 2022. We plan to enrol 261 adults and follow them for 9-months (6-month intervention period and a 3-month post-intervention follow-up period) to assess glycaemic control and other health indicators. A meta-analyses of clinical trials show a pooled difference of -0.6 percentage points in HbA1c after a minimum 8-12 weeks of exercise intervention ^12,13^. Consequently, the 6-month intervention period is designed to be sufficient to assess changes in lifestyle, but not of excess duration in order to reduce participant drop-out and loss to follow-up. If a 6-month trial of lifestyle intervention fails to adequately control blood glucose level, it may be appropriate to consider additional medication for glycaemic control ^4–6^.

Participants will be randomly assigned to one of three groups (in ratio 1:1:1): arm A (financial incentives in addition to standard care), arm B (financial and social incentives in addition to standard care), and arm C (control arm receiving standard care). All participants will establish a baseline step count of physical activity using the second week of recorded measurements. The first week of recorded measurements will be ignored to minimise the potential upward bias from elevated physical activity during initial device use. Subsequently, all participants will receive weekly increases of 1,000 daily step targets from their baseline levels to a maximum target of 10,000 steps per day (depending on age, physical condition, physician guidance). The 10,000-step target approximates the 30 minutes of daily exercise recommended by the American College of Sports Medicine ^14^. We will impute daily values less than 1,000 steps as these values are unlikely to represent actual activity ^15,16^. Participants will use their smartphones to track step counts on the same pre-specified activity tracking app for consistency. Although smartphones are expensive, smartphone penetration in Hong Kong is very high, at above 88% of the population aged 10 or above in 2017 ^17^. All participants will be provided with a pedometer to account for physical activity. Participants are followed for an additional 3-months after the cessation of incentives to assess any continued effects and the sustainability of lifestyle change.

Arm A (financial incentives): Participants will receive the usual standard care (same as control group), a loss-framed financial incentive, and weekly feedback on performance for 6 months.

Arm B (financial and social incentives): Participants will receive the usual standard care (same as control group), a loss-framed financial incentive, social incentives, and weekly feedback on performance for 6 months.

Arm C (control): Participants will receive standard care under the risk factor assessment and management programme (RAMP) including patient screening and education on diet, physical activity, and smoking conducted by a multi-disciplinary team ^18^.

The study will last for a total of 36 months including a 11-month suspension due to COVID-19 pandemic. This includes a 11-month recruitment period on rolling basis, 9-month follow-up period for each participant (consisting of 6-months intervention period and 3-month post-intervention period) and 3-month buffer for any rescheduling of follow-up. We plan to allow 4-months for the data analysis and 3-months to write-up our findings.

The pragmatic study design is based on the PRECIS-2 tool (Appendix 3) and RE-AIM frameworks.^19,20^ This trial is registered at ClinicalTrials.gov and will follow this pre-specified protocol.

## Participant enrolment

In this study, invitations to participate will be extended to community-dwelling adults in Hong Kong through general outpatient clinics (GOPC) for family medicine & primary care, District Health Centres and supporting organisations. Eligible and interested adults will be identified and invited to join this study during their routine medical appointment at the GOPC. The contact information of the patients will be forwarded to the research staff for screening and study meet-up arrangement. Participants will be screened over phone within one month. The patients will subsequently come to study clinics for enrolment and other trial-related activities.

## Informed consent

Written informed consent will be sought from each eligible adult who is willing to participate in the study – a copy of the consent form will be provided to the patient. We will assess the cognitive function and ability to provide informed consent using a screening tool (Mini-Cog^21^) for all participants age over 60 years.

## Enrolment interview

A short standardized questionnaire will be administered by trained research personnel to collect background information including demographics (age, sex, current job status, education level, monthly household income, smoking status), body weight and height, general functional and health status including breastfeeding (for women), existing medical conditions (chronic cardiovascular, pulmonary, renal, hepatic, metabolic, autoimmune and immunocompromising diseases or treatment), medication use, levels of physical activity, diet, and health-related quality of life.

## Collection of blood samples

Simple blood tests will be given to all participants as an incentive, and we will collect 3-10 mL of blood from each participant at baseline (if no contemporaneous results), 6-months and 9-months for this purpose. Only registered phlebotomist or trained nurses will collect blood samples. Blood specimens will be collected and tested by designated laboratory. Results will be issued by registered medical laboratory technologist.

## Randomization

The random allocation process will be concealed from the front-line team, using tamper-proof methods, by way of pre-assignment based on a computer-generated sequence of random numbers administered by an independent research assistant. A sequence of random numbers will be generated prior to the start of the study by a statistician. To avoid baseline unbalance, we will stratify the randomization by age (30-49, 50-70) and sex. Computer software (R) will be used to generate the randomization sequence, using a block randomization structure with block sizes of 6 for the enrolment of participants.

The study will remain blind to laboratory and analysis staff. Research staff members at the designated study clinics who had no access to the randomization sequence will allocate a unique code to each of the participants based on their order of attendance. Because the allocation process will be concealed, the identity of the intervention groups will be unknown to the laboratory staff. The randomization codes will be masked from those assessing the outcomes and will only be revealed to the investigators after completion of the follow-up. A study statistician, uninvolved with other trial activities, will have access to the randomization codes for the purpose of conducting regular interim analyses and safety monitoring.

We will enrol 261 participants and randomly allocate each participant to one of the three study arms in equal proportion (1:1:1 ratio) (Figure 1). We assume 10% of participants do not complete the intervention or are lost to follow-up.

**Figure 1. CONSORT Flow Diagram**

Discontinued or Lost to follow-up (n=13)

Analysed (n=74)

Analysed (n=74)

**Analysis**

Analysed (n=74)

Discontinued or Lost to follow-up (n=13)

Discontinued or Lost to follow-up (n=13)

**Follow-Up**

**Allocation**

**Arm A: Financial incentives**

- Received allocated control (n=87)

**Arm B: Financial and social incentives**

- Received allocated intervention (n=87)

**Arm C: Control**

- Received allocated intervention (n=87)

**Enrolment**

Assessed for eligibility (n=301)

Excluded (n=40)

- Not meeting inclusion criteria
- Declined to participate
- Other reasons

Randomized (n=261)

## Follow-up assessments

A follow-up assessment on health information, similar to the baseline enrolment questionnaire, including body weight and height, general health, medication usage and health service usage will be collected when participants have blood drawn 6 months after enrolment. In usual practice, newly diagnosed patients with type 2 diabetes attend regular medical appointment every 3 months to monitor their condition. A brief follow-up assessment will be completed at 9 months for all participants to monitor changes in health status of the participants longitudinally.

## Incentives

Incentives will be provided in this study to compensate participants for their time, inconvenience, and discomfort, which is standard research practice for studies of this nature, and we believe is necessary to maintain participation. All participants will be offered an incentive of free basic blood tests including glucose control and cholesterol at enrolment (if no contemporaneous results), 6-months and 9-months. Laboratory test results will be given back to each participant. If a result falls out of its reference range, our research team will advise the participant to seek medical consultation for further assessment. All participants will also be provided with a pedometer to track their physical activity. In addition, they will be offered an incentive of a gift voucher valued at HKD 50 gift coupon upon: (1) completion of study enrolment and (2) completion of the study. Participants in the two intervention groups will receive up to HKD 1,000 cash as the intervention being administered.

# Selection and Withdrawal of Subjects

## Subject inclusion criteria

- Chinese adults aged 30-70 years.
- Capable of providing informed consent.
- Resident in Hong Kong.
- Able to communicate in English or Chinese.
- Diagnosed with type 2 diabetes within past year.
- 6.5% ≤ HbA1c ≤ 9%.
- Willing to take blood tests.
- Access to a smartphone to track physical activity and receive text messages.
- Physically mobile for duration of the trial.

The inclusion criteria of HbA1c ≤ 9% is in accordance with the Hong Kong reference framework for diabetes care recommendation of insulin for HbA1c > 9%^5^.

## Subject exclusion criteria

- Planned surgery in the next 6 months.
- Biological impairment or health condition affecting the ability to walk, such as blindness, physical immobility, and paralysis.
- Pregnant or breastfeeding.
- On insulin for diabetes control.

## Subject withdrawal criteria

Participants are free to withdraw their consent and participation at any stage of the study with no explanation and without any prejudice. If the participant chooses to withdraw, the reasons for withdrawal and participant health status will be recorded. Participants who pass away during the study period will be identified and recorded through interview with the participant’s family member or friend, or identified from hospital records or death certificates, and abstracted from death certificates from Deaths Registry at the end of the study.

# Interventions to be Administered

All participants will receive standard care including patient screening and education on diet, physical activity, and smoking conducted by a multi-disciplinary team ^18^. Arm A (financial intervention) will receive (loss-framed) financial incentives, in addition to weekly feedback on performance for 6 months; Arm B (financial and social interventions) will receive (loss-framed) financial incentives and social incentives, in addition to weekly feedback on performance for 6 months; Arm C (control) will receive no additional intervention during the trial.

The financial incentives use a loss-framed design based on the motivation of loss aversion and the endowment effect. Loss-framed incentives are more effective at increasing physical activity compared to lottery-framed incentives or gain-framed incentives ^22^. Participants randomized to the intervention arms are credited with HKD 1,000 in their viewable virtual account at the beginning of the intervention for the endowment effect during the first two weeks. After baseline step counts are gathered in the second week, participants will lose HKD 40 for each subsequent week of non-adherence to physical activity. Non-adherence is defined as not meeting their personal weekly step target. The amount of financial incentives is small compared to the expected direct medical cost of diabetes to the public sector in Hong Kong of HKD 11,919 (USD 1,521) per year for a person without any complications ^23^.

The social incentives are peer comparison and social support. Since the participants have personalised target step counts, we will not compare direct step counts but rather assess whether they met or exceeded their target. We will feedback the percentage of participants who met or exceeded their targets. We expect the periodic reminders will lead participants to conform their behaviour closer to the social norm. For social support, we will share the participants’ weekly performance with their nominated supporter to act as a social incentive. The nominated supporter is anticipated to encourage the participants to meet their target.

Feedback messages will be positively framed and sent weekly to garner encouragement. Participants enrolled in intervention arm A (financial incentives) with their daily step count target met will receive individualized feedback by text message: “Well done and congratulations! You achieved your physical activity goal this week! You have $1,000 remaining in your account.”. The remaining participants in this arm will be told the balance in their account after penalty of losing HKD40: “Sorry, you did not meet your physical activity goal this week. You lost $40 from your account. Remaining balance = $960.”. Participants achieving their daily step-count target in intervention arm B (financial and social incentives) will receive weekly monitoring and individualized feedback during the incentive period by text message: “Well done and congratulations! You achieved your physical activity goal this week! X% of all participants met their targets this week (X% exceeded!). You have $1,000 remaining in your account.”. Those not meeting their targets in this arm will be told they did not meet their targets and the balance in their account after penalty of losing HKD40: “Sorry, you did not meet your physical activity goal this week. X% of all participants met their targets this week (X% exceeded!). You lost $40 from your account. Remaining balance = $960. Keep it up and strive for your best next week!”.

Participants who are not able to record and share their step counts on a weekly basis will receive messages or phone calls from the research team to record their physical activity performance. The incentives are discontinued during the post-intervention follow-up period.

Participants being enrolled in the control arm will not be told their baseline step count after the second week. They will receive the routine care but not weekly feedback nor messages.

## Medications permitted and not permitted before and/or during the study

Participants eligible for the study must be newly diagnosed with diabetes within a year. Participants can continue taking other medication, treatment or routine for conditions other than diabetes should they be enrolled at any time of the study. Participants are allowed to initiate additional medication for glycaemic control during the 6-month follow-up period, and other medication including agents to control lipid levels or blood pressure. The inclusion criteria of HbA1c ≤ 9% is in accordance with the Hong Kong reference framework for diabetes care recommendation of insulin for HbA1c > 9%^5^.

## Stopping rule

Given that the study interventions are non-invasive and non-clinical, we do not plan to conduct any interim analyses to inform whether to continue the study. However, if we observe any SAEs confirmed by a physician where appropriate, we will terminate participation for the participant and conduct an immediate investigation while suspending the trial until a satisfactory resolution.

# Outcome Measures

## Primary outcome

Glycaemic control defined as the change in HbA1c levels at 6-months (and 9-months) compared to baseline levels. HbA1c levels reflect the average glucose level over the past 2-3 months (not influenced by daily fluctuations) and has strong predictive value for diabetes complications^5^.

## Secondary outcomes

The secondary outcome measures at 6-months and 9-months include:

- proportion of participants with good/excellent diabetes control (HbA1c threshold ≤7% & ≤6.5%)
- change in body weight and body mass index
- changes in lipid profile (HDL- and LDL-cholesterol) and blood pressure
- proportion of participants requiring initiation of additional medication or higher dose adjustment for glycaemic control
- proportion of smokers quitting
- change in level of physical activity (step count)
- change in health care attendances and admissions

## Methods for assessing and analysing of efficacy parameters

Blood samples at baseline, 6-months and 9-months will be tested at an accredited laboratory for blood glucose (fasting glucose, HbA1c), lipid profile (total cholesterol, LDL-cholesterol, HDL-cholesterol, triglycerides), routine blood profile (blood cell counts) and urine cotinine (if smoker).

For objective measurement of step counts, all participants will receive a pedometer to account for physical activity not measured by the smartphone (e.g. when smartphone left on a table). Although smartphones are expensive, smartphone penetration in Hong Kong is very high, at above 88% of the population aged 10 or above in 2017 ^17^. Participants will use their smartphones to track step counts using the same pre-specified activity tracking app to ensure consistency.

Health-related physical activity (Metabolic Equivalent of Task/MET-minutes per week) will be also assessed using participant responses to the validated International Physical Activity Questionnaires (IPAQ)^24^. Adherence to physical activity targets is measured as the mean proportion of participant-weeks achieving their weekly physical activity goals based on step counts. Measurements may also be compared to results from their usual diabetes clinic visits (if any). We will administer EQ-5D questionnaires to measure the health status of the participants and quality of life ^25^, and food frequency questionnaire to evaluate the diet of patients. Surveys will be administered at enrolment, 6-months and 9-months.

# Assessment of Safety

Participants should report any adverse events to the study co-ordinator (contact details are provided to participants). Given the non-clinical nature of the intervention, we do not anticipate any occurrence of SAEs within our study. We will survey participants for any safety issues at 6-months and 9-months after enrolment. We will record and inform the IRB of any reported adverse events. For stopping rule, refer to section 6.2 for details. All trial-related activities including enrolment and follow-up will be conducted by our delegated research team members from the School of Public Health, the University of Hong Kong.

# Statistical analysis

## Statistical methods

We compare sample characteristics between the arms using ANOVA for continuous variables, and Kruskal-Wallis test for categorical variables. The change from baseline HbA1c will be calculated for each individual participant. The time-matched change from baseline data will be analysed by a repeated-measure analysis of covariance (ANCOVA), fitting terms appropriate to the study design, including treatment group, time and group-by-time interaction. Participants will be included in the model as a random effect. Baseline values will be included as a covariate in the above models. Interim analysis will be performed after ~50% participants having completed the study. We will use the bootstrap method to resample participant groups 200 times to calculate the 95% confidence intervals. As a sensitivity analysis, we will use per protocol analysis to check for the robustness of our results. All analyses will be conducted using R statistical software.

## Procedure for accounting for any missing data

If missing data are present in the computerized database, we will first check the original records to attempt to complete those variables. In the analyses, we will use multiple imputation^26^ with 10 imputed datasets to replace missing values on outcome and predictor variables (using the *mice*/*Hmisc* package in R) and combine the results using Rubin’s rules^27^. If 10 imputed datasets are not sufficient to ensure stability of estimates, we will use 20 imputed datasets. The imputation predictors will include HbA1c at baseline, 6-months and 9-months; weight; age; and sex. Multiple imputation makes maximum use of available data and maximizes statistical power while requiring less strict theoretical assumptions than to a complete case analysis, or single imputation of mean values; this is a standard method for analysing clinical trials data ^28^.

## Procedure for reporting any deviations from the original statistical plan

Any deviations from the original statistical plan will be described and justified in the final report.

## Selection of participants to be included in the analyses

We will follow an intention-to-treat approach in the analyses; therefore all randomized participants will be included.

## Sample size justification

We propose to enrol 261 participants into our study. Based on published meta-analyses of clinical trials on the effect of lifestyle modification (diet and exercise interventions), we assume a mean difference of -0.6 in HbA1c with a standard deviation for each group of 1.5 percentage points.^12,13^ A sample size of 234 subjects (78 per group) will achieve 80% power to reject the null hypothesis of equal means between any two groups. The calculation is based on equal-variance t-test with a significance level of 0.05. We allow for a 10% dropout rate, giving the total target sample size of 261 (87 per group). Interim analysis will be performed after ~50% participants having completed the study. Sample size re-estimation may be applied based on interim look results upon the advice of our biostatistician. ​

# Direct Access to Source Data

The investigator(s)/institution(s) will permit trial-related monitoring, audits, IRB/REC review, and regulatory inspection(s), and provide direct access to source data/documents.

# Quality Control and Quality Assurance

Our research personnel will follow Good Clinical Practice (GCP) and our standard operating procedures to ensure proper conduct throughout the study. The trial will be conducted in strict compliance with the approved study protocol. Only trained phlebotomists or registered/enrolled nurses will collect blood samples. Other research personnel will also be trained and briefed to collect data, recruit participants, and answer questions from the participants or their family members.

# Ethics

The study will be conducted in compliance with this protocol, the Declaration of Helsinki ^29^, Good Clinical Practice, and the applicable regulatory requirements. In line with Good Clinical Practice guidelines, a Data Monitoring Committee will be established as an independent group consisting of biostatistician, clinician and person with expertise appropriate to the study at hand. The Data Monitoring Committee will meet periodically before and during the study to review data on safety and outcomes. Written informed consent will be obtained by our research staff directly from eligible participants after thoroughly explaining the study design, purpose and necessity of randomization to each potential participant. Rights and well-being of participants will be well protected during the whole study period, participation in the study is on a purely voluntary basis and participants are free to withdraw their consent and participation at any stage of the study with no explanation and without any prejudice.

Participants in the control arm will receive usual care. There are no major risks associated with the interventions. The process of collecting blood is associated with minimal risk, a small number of participants may have local reactions with the symptom of mild transient pain and bruising, but this will not influence their health. Another potential risk is loss of confidentiality due to data breach, but as the intervention and general physiological metrics do not carry large social stigma, these costs would be limited, and we will make every effort to control the risk. Participants in the study, as members of the whole society, will also benefit indirectly from this trial as the findings will make important contributions to our knowledge regarding public health strategies and inform evidence-based health policy for chronic diseases in Hong Kong, China, and elsewhere.

# Data Handling and Record Keeping

Qualtrics is a software application and workflow methodology designed to collect and manage data for research studies. Qualtrics is a secure, web-based applications designed to support data capture for research studies, providing: 1) an intuitive interface for validated data entry; 2) audit trails for tracking data manipulation and user activity; 3) automated export procedures for data downloads to Excel, PDF, and common statistical packages (SPSS, SAS, Stata, R); and 4) procedures for importing data from external sources.

Data for the questionnaire will be entered by the research staff directly to the Qualtrics electronic data capture system at the time of contact with the participants. All data obtained from the participants will be extracted into the secure Qualtrics database. This extraction will be facilitated by authorized researchers. The servers supporting the project database are secured by the University of Hong Kong in password-protected computers. All records will be entered using a personal identification code, making the records unidentifiable in Qualtrics. The link between the protected health information and personal identification code will be stored separately. All identifying data is anticipated to remain on the University of Hong Kong Qualtrics server in a locked cabinet in a locked room. Personal data will be protected in the database such that the personal data cannot be downloaded when the other anonymized study data are downloaded for analysis. The University of Hong Kong will have quality control check, tracking and cleaning of the data every week. Only fully deidentified data will be shared with researchers outside the study.

All data will be stored in the School of Public Health, HKU. All personal data will be kept confidential. Data from this study may be used in presentations and papers. Any potentially identifying information such as visit dates will not be shown.

# Financing

This study is supported by a GRF grant from the Research Grants Council of the Hong Kong Special Administrative Region, China.

# Publication Policy

The study will be published in international peer-reviewed journals. The results will be reported according to CONSORT guidelines.

# Supplements

Appendix 1: Informed consent form (bilingual)

Appendix 2: Participant information sheet (English and Chinese)

Appendix 3: PRECIS-2 score sheet

# References

1. Quan J, Li TK, Pang H, Choi CH, Siu SC, Tang SY, et al. Diabetes incidence and prevalence in Hong Kong, China during 2006–2014. Diabet Med. 2017;34(7):902–8.

2. Wang L, Gao P, Zhang M, Huang Z, Zhang D, Deng Q, et al. Prevalence and Ethnic Pattern of Diabetes and Prediabetes in China in 2013. JAMA. 2017 Jun 27;317(24):2515–23.

3. Centre for Health Protection. Statistics on behavioural risk factors [Internet]. 2018 [cited 2018 Oct 16]. Available from: https://www.chp.gov.hk/en/static/24016.html

4. American Diabetes Association. 5. Prevention or Delay of Type 2 Diabetes: Standards of Medical Care in Diabetes—2018. Diabetes Care. 2018 Jan;41(Supplement 1):S51–4.

5. Food and Health Bureau. Hong Kong Reference Framework for Diabetes Care for Adults in Primary Care Settings [Internet]. Hong Kong SAR; 2018 [cited 2018 Oct 15]. Available from: http://www.pco.gov.hk/english/resource/professionals_diabetes_pdf.html

6. NICE. Diabetes in adults: management NICE guidelines (NG28). 2015. 2015.

7. Volpp KG, Asch DA, Galvin R, Loewenstein G. Redesigning Employee Health Incentives — Lessons from Behavioral Economics. N Engl J Med. 2011 Aug 4;365(5):388–90.

8. Kahneman D, Knetsch JL, Thaler RH. Anomalies: The Endowment Effect, Loss Aversion, and Status Quo Bias. J Econ Perspect. 1991 Mar;5(1):193–206.

9. Kahneman D, Tversky A. Prospect Theory: An Analysis of Decision under Risk. Econometrica. 1979;47(2):263–91.

10. Kullgren JT, Hafez D, Fedewa A, Heisler M. A Scoping Review of Behavioral Economic Interventions for Prevention and Treatment of Type 2 Diabetes Mellitus. Curr Diab Rep. 2017 Jul 28;17(9):73.

11. Hsee CK, Weber EU. Cross-national differences in risk preference and lay predictions. J Behav Decis Mak. 1999 Jun;12(2):165–79.

12. Boulé NG, Haddad E, Kenny GP, Wells GA, Sigal RJ. Effects of Exercise on Glycemic Control and Body Mass in Type 2 Diabetes Mellitus: A Meta-analysis of Controlled Clinical Trials. JAMA. 2001 Sep 12;286(10):1218–27.

13. Thomas D, Elliott EJ, Naughton GA. Exercise for type 2 diabetes mellitus. Cochrane Database Syst Rev [Internet]. 2006 [cited 2018 Oct 19];(3). Available from: http://www.cochranelibrary.com/cdsr/doi/10.1002/14651858.CD002968.pub2/abstract

14. Goff DC, Lloyd-Jones DM, Bennett G, Coady S, D’Agostino RB, Gibbons R, et al. 2013 ACC/AHA Guideline on the Assessment of Cardiovascular Risk: A Report of the American College of Cardiology/American Heart Association Task Force on Practice Guidelines. Circulation. 2014 Jun 24;129(25 suppl 2):S49–73.

15. Bassett DR, Wyatt HR, Thompson H, Peters JC, Hill JO. Pedometer-measured physical activity and health behaviors in U.S. adults. Med Sci Sports Exerc. 2010 Oct;42(10):1819–25.

16. Kang M, Zhu W, Tudor-Locke C, Ainsworth B. Experimental Determination of Effectiveness of an Individual Information-Centered Approach in Recovering Step-Count Missing Data. Meas Phys Educ Exerc Sci. 2005 Oct;9(4):233–50.

17. Census and Statistics Department. Thematic Household Survey Report - Report No. 64 [Internet]. Hong Kong SAR; 2018 [cited 2018 Oct 18]. Available from: https://www.censtatd.gov.hk/hkstat/sub/gender/itu/index.jsp

18. Fung CS, Chin WY, Dai DS, Kwok RL, Tsui EL, Wan YF, et al. Evaluation of the quality of care of a multi-disciplinary risk factor assessment and management programme (RAMP) for diabetic patients. BMC Fam Pract. 2012 Dec 5;13:116.

19. Loudon K, Treweek S, Sullivan F, Donnan P, Thorpe KE, Zwarenstein M. The PRECIS-2 tool: designing trials that are fit for purpose. BMJ. 2015 May 8;350(may08 1):h2147–h2147.

20. Glasgow RE, Harden SM, Gaglio B, Rabin B, Smith ML, Porter GC, et al. RE-AIM Planning and Evaluation Framework: Adapting to New Science and Practice With a 20-Year Review. Front Public Health. 2019 Mar 29;7:64.

21. Borson S, Scanlan JM, Chen P, Ganguli M. The Mini-Cog as a Screen for Dementia: Validation in a Population-Based Sample: MINI-COG IN MOVIES. J Am Geriatr Soc. 2003 Oct;51(10):1451–4.

22. Patel MS, Asch DA, Rosin R, Small DS, Bellamy SL, Heuer J, et al. Framing Financial Incentives to Increase Physical Activity Among Overweight and Obese Adults: A Randomized, Controlled Trial. Ann Intern Med. 2016 Mar 15;164(6):385.

23. Jiao F, Wong CKH, Tang SCW, Fung CSC, Tan KCB, McGhee S, et al. Annual direct medical costs associated with diabetes-related complications in the event year and in subsequent years in Hong Kong. Diabet Med. 2017;34(9):1276–83.

24. Craig CL, Marshall AL, Sjorstrom M, Bauman AE, Booth ML, Ainsworth BE, et al. International physical activity questionnaire: 12-country reliability and validity. Med Sci Sports Exerc. 2003;35(8):1381–95.

25. Gusi N, Olivares PR, Rajendram R. The EQ-5D Health-Related Quality of Life Questionnaire. In: Preedy VR, Watson RR, editors. Handbook of Disease Burdens and Quality of Life Measures [Internet]. New York, NY: Springer New York; 2010 [cited 2020 Jan 20]. p. 87–99. Available from: http://link.springer.com/10.1007/978-0-387-78665-0_5

26. Schafer JL. Multiple imputation: a primer. Stat Methods Med Res. 1999 Feb;8(1):3–15.

27. White IR, Royston P, Wood AM. Multiple imputation using chained equations: Issues and guidance for practice. Stat Med. 2011 Feb 20;30(4):377–99.

28. Molenberghs G, Kenward MG. Missing data in clinical studies. Reprinted. Chichester: Wiley; 2008. 504 p. (Statistics in practice).

29. World Medical Association Declaration of Helsinki: Ethical Principles for Medical Research Involving Human Subjects. JAMA. 2013 Nov 27;310(20):2191.
